# Evaluation and validation of reference genes for RT-qPCR gene expression in *Naegleria gruberi*

**DOI:** 10.1038/s41598-023-43892-3

**Published:** 2023-10-05

**Authors:** Tania Martín-Pérez, Martina Köhsler, Julia Walochnik

**Affiliations:** Institute of Specific Prophylaxis and Tropical Medicine, Center for Pathophysiology, Infectiology and Immunology, Medical University of Vienna, 1090 Vienna, Austria

**Keywords:** Bioinformatics, Reverse transcription polymerase chain reaction, Gene expression, Parasitology

## Abstract

*Naegleria gruberi* is a free-living amoeboflagellate commonly found in freshwater and in soils around the world. It is a non-pathogenic relative of *Naegleria fowleri*, which is the etiologic agent of Primary Amoebic Meningoencephalitis (PAM). PAM occurs world-wide and it is considered a rare disease, but its fatality rate is high (96%) mainly because of delay in initiation of treatment due to misdiagnosis and lack of a specific treatment. The analysis of gene expression by quantitative real-time PCR in *N. gruberi* could be a highly efficient means to understand the pathogenicity of *N. fowleri* and also to find drug targets. Accurate RT-qPCR analysis requires correct normalization of gene expression data using reference genes (RG), whose expression should be constant under different experimental conditions. In this study, six genes, representing the most frequently used housekeeping genes, were selected for evaluation as reference genes in *N. gruberi*. The expression and stability of these genes was evaluated employing four algorithms (geNorm, NormFinder, BestKeeper and RefFinder). This work shows significant variations of the stability of RGs depending on the algorithms employed and on the experimental conditions (i.e. logarithmic, stationary, heat-shock and oxidative stress). The geNorm, NormFinder and RefFinder analysis of all the experimental conditions in combination revealed that *ACT* and *G6PD* were the most stable RGs. While BestKeeper analysis showed that *18S* and *TBP* were the most stable RGs. Moreover, normalization of *HSP90* gene expression with the most stable RGs resulted in an upregulation whereas when the normalization was done with the unstable RGs, the gene expression was not reliable. Hence, the implications of this study are relevant to gene expression studies in *N. gruberi*.

## Introduction

*Naegleria gruberi* is a free-living amoeba of the genus *Naegleria,* which occurs worldwide in wet soil and bodies of freshwater, such as lakes, ponds, swimming pools, spas and also wastewater sewage^1,2^. *N. gruberi* is being studied to answer questions on early eukaryotic evolution^3^. Moreover, *N. gruberi* is a safe model organism for its pathogenic relative *N. fowleri*^4^*,* because of a similar genetic and biochemical repertoire^5^. *N. fowleri* is the only species in the *Naegleria* genus known to produce an acute fulminant, necrotizing, and hemorrhagic meningoencephalitis called Primary Amoebic Meningoencephalitis (PAM). PAM is considered a rare disease that is underreported^6,7^. In the past years, an increase in the number of reported cases has been observed, mostly due to raised awareness of the disease and better diagnostic tools. PAM has a very high fatality rate, around 96%, and most cases occur in healthy children following recreational water activities^6^. Recently, cases related to the ablution rituals practiced by Muslims have been described, especially when cleansing the nose using so-called neti pots^8^. The main reason for the high fatality rate is delayed diagnosis, leading to delayed initiation of treatment. In addition, there is a lack of specific treatment related to many biological mechanisms of *Naegleria* being unknown, such as the factors that contribute to its pathogenicity, the components of the redox chain, and its mechanism to transform into cysts or to the flagellar form^9^. To understand all these mechanisms, it is necessary to evaluate the expression of the respective genes, with real-time quantitative reverse transcription polymerase chain reaction (RT-qPCR) being the method of choice for the quantification of mRNA^10^. The RT-qPCR is a powerful and accurate tool that can provide reliable and reproducible results, but it is important to select a robust normalization approach. The most common and simple method for normalizing the mRNA levels of a target gene is comparing them with reference genes (RGs), whose expression levels are constant under different experimental conditions^11^. Nevertheless, several studies have highlighted there is no single gene that fulfills the criteria required from a universal RG because its expression stability varies depending on the conditions or the type of cell^12,13^. Classic RGs or housekeeping genes (for example β*-actin*, *glyceraldehyde-3-phosphate dehydrogenase*, *18S ribosomal RNA*) have been used as references in Northern blots, RNase protection assays, and conventional RT-PCR assays^14^. It has been proven that the expression levels of these classic RGs can vary greatly and, in some experimental conditions, are invalid for normalization and could lead to biased findings^11^. As far as we know, in the numerous studies that have investigated changes in gene expression in the genus *Naegleria*, none have demonstrated the stability, suitability, and reliability of RGs for qRT-PCR standardization^15–18^.

This research assesses the stability of potential RGs for normalizing gene expression in *N. gruberi* using RT-qPCR across diverse conditions. The *18S rRNA gene* (*18S*), *actin* (*ACT*), *glucose-6-phosphate dehydrogenase* (*G6PD*), *glycerol-3-phospahte dehydrogenase* (*GAPDH*), *hypoxanthine–guanine phosphoribosyltransferase* (*HPRT*), and *TATA-binding-protein* (*TBP*) genes were evaluated under different experimental conditions, such as heat-shock and exposure to hydrogen peroxide. The RGs were analyzed using four algorithms: geNorm^19^, NormFinder^20^, BestKeeper^21^ and RefFinder^22^.

## Results

### Primer specificity and the amplification efficiency

Each primer set of RGs produced a single amplicon in agarose gel electrophoresis (Supplementary Fig. S1). The amplification efficiency and R^2^ of the RT-qPCR assays of RGs were calculated with the slope of the standard curve. The amplification efficiency ranged from 95 to 122%. The primers of each RG with the best amplification efficiency were selected for further analysis (Table 1). The amplification efficiency and coefficients of determination (R^2^) of the primer set selected ranged from 95 to 105% and 0.948 to 0.999, respectively.

**Table 1:** Primer details and parameters derived from a tenfold standard curve of the six references genes and target gene. Underlined primers were chosen for further analysis.

Primer	Primer sequence (5’–3’)	Amplicon length (bp)	Average Tm (°C)	Amplification efficiency (%)	Slope	Correlation coefficient (R^2^)	GenBank accession no
18S (1)	F: GCCTGAGAAATCGCTACCAC R: CAGAAGACAATACCTCCCCAC	125	60.1	94.80	− 3.50	0.949	M18732.1
18S (2)	F: ATAACGAACGAGACCTAAGCC R: TCCGACAAACTAACCCTTCCC	70	62.5	95.96	− 3.43	0.948	
ACT (1)	F: CCTCGTGCTGTTTTCCCATC R: GCTTCATCTCCGACATAGGC	95	57.2	105.95	− 3.19	0.998	XM_002672078.1
ACT (2)	F: TGGAATGGAAGCTGCTGGTA R: GGTTGTACCGCCTGAAAGTA	111	56.8	122.60	− 2.88	0.976	
G6PD (1)	F: GTTCAACCAGAGCCATATCC R: TGCTTGCTACTAAACCATAACG	84	56.9	102.94	− 3.26	0.991	XM_002681275.1
G6PD (2)	F: GGGAGTTGAAGGAAGAGGTG R: GTTCCATAGCAATGAGAGCC	101	55.0	101.50	− 3.29	0.999	
GAPDH (1)	F: TGTCCACGCTGTTACTGCTA R: ATTGTAACCAGCAGCACGAC	84	56.9	96.78	− 3.44	0.986	XM_002669943.1
GAPDH (2)	F: TGGTCGTGAAATCCACGTTT R: TAGCACCACCCTTCAAGTGC	140	59.2	101.34	− 3.29	0.999	
HPRT (1)	F: CTTGTGTCTTGTTGACTGCCC R: CCAAACCGTATCCAACAACGA	107	60.7	117.45	− 2.97	0.976	XM_002673756.1
HPRT (2)	F: CGTGTCAAGGAATTGGCTCA R: ACCCTTCAAAACTGGAACCA	93	57.0	104.44	− 3.23	0.998	
TBP (1)	F: ACGGTAAATCTTGCTTGCGA R: ACGCATAATCACAGCAGCAA	98	55.8	105.11	− 3.21	0.999	XM_002679885.1
TBP (2)	F: GACACCAGTGCCAGGTACAC R: GAAGAGGTGTTGATGTCGGC	101	58.4	99.44	− 3.35	0.998	
Target gene							
HSP90 (1)	F: TCTAATAGACTCTCCTCTGCAC R: TTCATCATCCAATCCATACCAC	127	57.1	100.54	− 3.31	0.997	XM_002682845.1
HSP90 (2)	F: AATAGACTCTCCTCTGCACC R: TTCATCATCCAATCCATACCAC	124	55.1	97.05	− 3.40	0.999	

### Quantification cycle (Cq) values and the expression profiles of the six RGs

**Figure 1:**
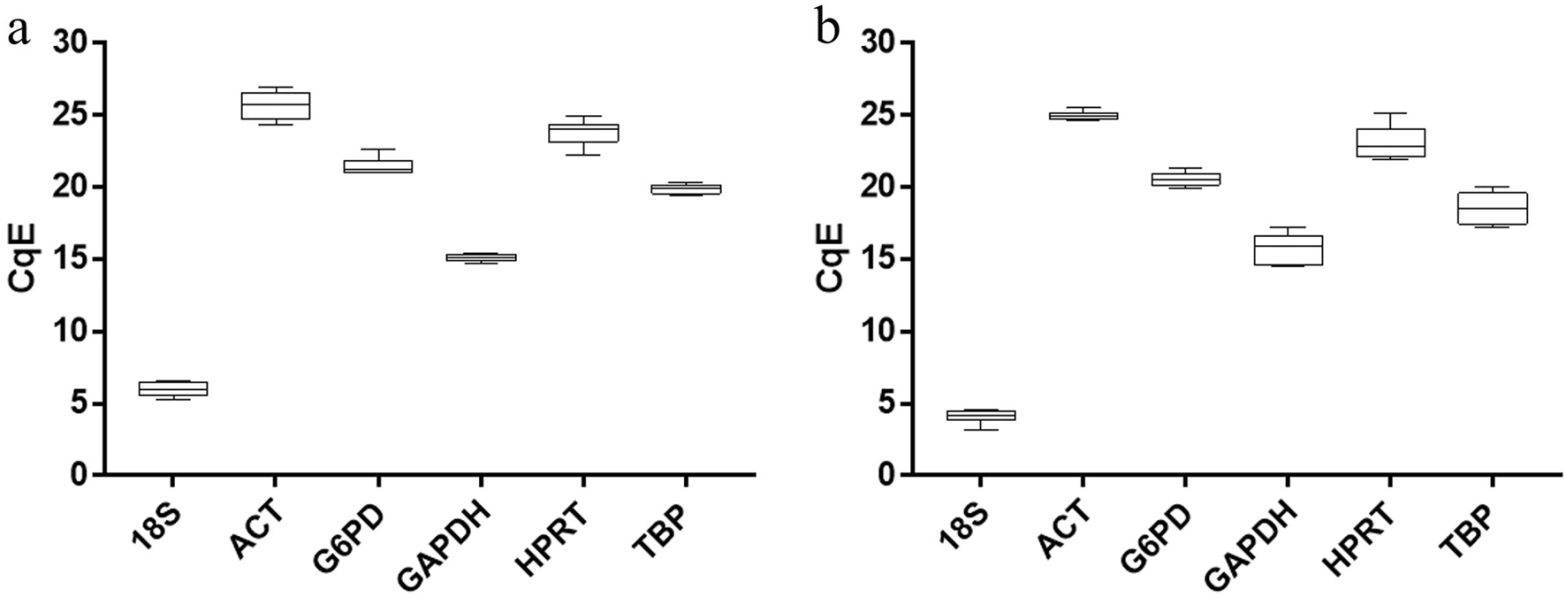
Box plot of mean Cq values after efficiency correction (CqE) of the RGs from two experimental conditions. (**a**) Different growth stage (LOG and STAT). (**b**) Stressful conditions (HS and OS). The average (horizontal line), upper and lower quartiles (box), and maximum and minimum values (whisker) of each RG are shown.

RT-qPCR assays were performed with the selected primers for the six candidate RGs using RNA as a template extracted from different *N. gruberi* growth-phase conditions (logarithmic and stationary) and under stressful conditions (heat-shock and oxidative stress). Based on the Cq values obtained from RT-qPCR (Supplementary Table S1), the efficiency correction (CqE) was calculated and is displayed in a box diagram (Fig. 1 and Supplementary Table S2). The average Cq values of the RGs ranged from 5.98 to 26.23 and 4.12 to 24.95 for growth stages and stressful conditions, respectively. The results reveal that *GAPDH* had the smallest variation, followed by *TBP*, whereas *ACT* had the largest variation for different growth phases. In stress conditions, the smallest variation was observed in *ACT*, followed by *18S*, whereas *HPRT* displayed the largest variation. Regardless of expression levels, *18S* was the gene with the highest expression (lower CqE value), and *ACT* was the least expressed (higher CqE value) in both experimental conditions.

### Stability of the candidate RGs

**Figure 2:**
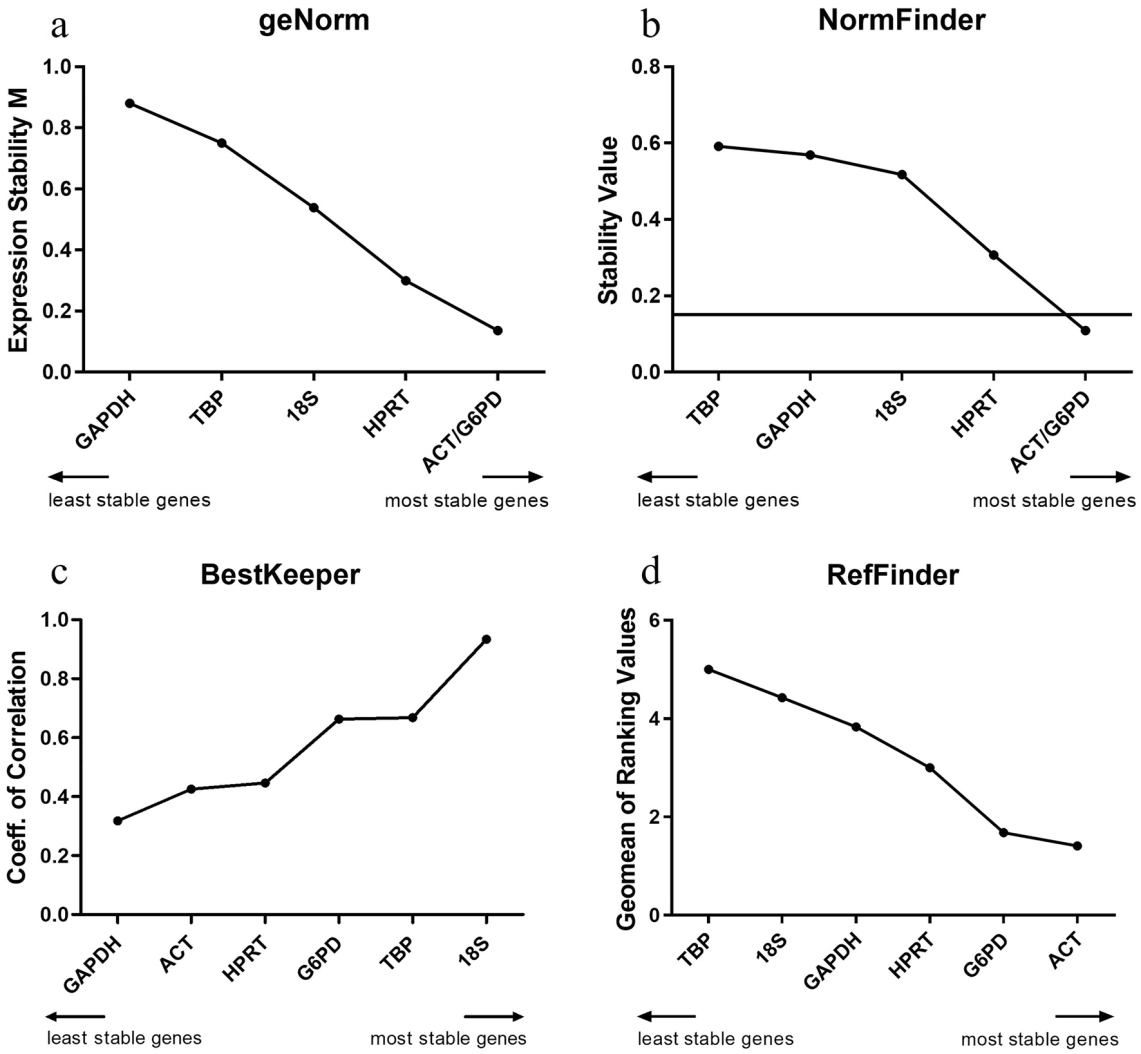
Expression stability of RGs for the normalization of all conditions in *N. gruberi* cells calculated by different algorithms. (**a**) geNorm expression stability M. (**b**) NormFinder stability values, the line indicates the NormFinder cut-off value of 0.15. (**c**) BestKeeper coefficient of correlation. (**d**) RefFinder geomean of ranking values.

Four algorithms were used to calculate the expression stability of RGs under different experimental conditions (i.e., logarithmic, stationary, heat-shock, and oxidative stress). The outcomes of all the analyses revealed the rankings of the RGs varied depending on the conditions tested (Table 2, Supplementary Table S3). Across all conditions combined (Fig. 2), *ACT* and *G6PD* were ranked equally as most stable, with geNorm and NormFinder. In the RefFinder analysis, *ACT* and *G6PD* were also the most stable RGs, but *ACT* ranked first. However, the analysis with BestKeeper was remarkably different; *18S* and *TBP* were the most stable RGs.

**Table 2:** Ranking of RGs for all conditions of *N. gruberi* cultures based on geNorm, NormFinder, BestKeeper and RefFinder.

	Rank	geNorm	NormFinder	BestKeeper	RefFinder
AC	1	ACT/G6PD	ACT/G6PD	18S	ACT
	2			TBP	G6PD
	3	HPRT	HPRT	G6PD	HPRT
	4	18S	18S	HPRT	GAPDH
	5	TBP	GAPDH	ACT	18S
	6	GAPDH	TBP	GAPDH	TBP
GP	1	G6PD/GAPDH	HPRT	18S	HPRT
	2		G6PD	GAPDH	G6PD
	3	ACT	TBP	TBP	GAPDH
	4	HPRT	GAPDH	G6PD	ACT
	5	TBP	ACT	HPRT	TBP
	6	18S	18S	ACT	18S
SC	1	18S/ACT	GAPDH	G6PD	ACT
	2		G6PD	18S	G6PD
	3	G6PD	ACT	GAPDH	18S
	4	HPRT	18S	HPRT	GAPDH
	5	GAPDH	HPRT	TBP	HPRT
	6	TBP	TBP	ACT	TBP
LOG	1	18S/GAPDH	G6PD/TBP	TBP	TBP
	2			ACT	G6PD
	3	TBP	HPRT	18S	GAPDH
	4	G6PD	GAPDH	GAPDH	18S
	5	HPRT	18S	G6PD	HPRT
	6	ACT	ACT	HPRT	ACT
STAT	1	18S/GAPDH	18S/GAPDH	G6PD	GAPDH
	2			18S	18S
	3	TBP	TBP	TBP	TBP
	4	ACT	ACT	HPRT	ACT
	5	G6PD	G6PD	GAPDH	G6PD
	6	HPRT	HPRT	ACT	HPRT
HS	1	TBP/G6PD	TBP	GAPDH	TBP
	2		G6PD	TBP	G6PD
	3	GAPDH	ACT/GAPDH	G6PD	18S
	4	18S		18S	ACT
	5	ACT	18S	HPRT	GAPDH
	6	HPRT	HPRT	ACT	HPRT
OS	1	ACT/HPRT	G6PD	18S	ACT/G6PD
	2		TBP	G6PD	
	3	G6PD	ACT	TBP	HPRT
	4	18S	18S	HPRT	TBP
	5	TBP	HPRT	GAPDH	18S
	6	GAPDH	GAPDH	ACT	GAPDH

*AC* All conditions combined, *GP* Growth phases: LOG + STAT, *SC* Stress conditions: HS + OS, *LOG* Logarithmic phase, *STAT* Stationary phase, *HS* Heat shock, *OS* Oxidative stress.

**Figure 3:**
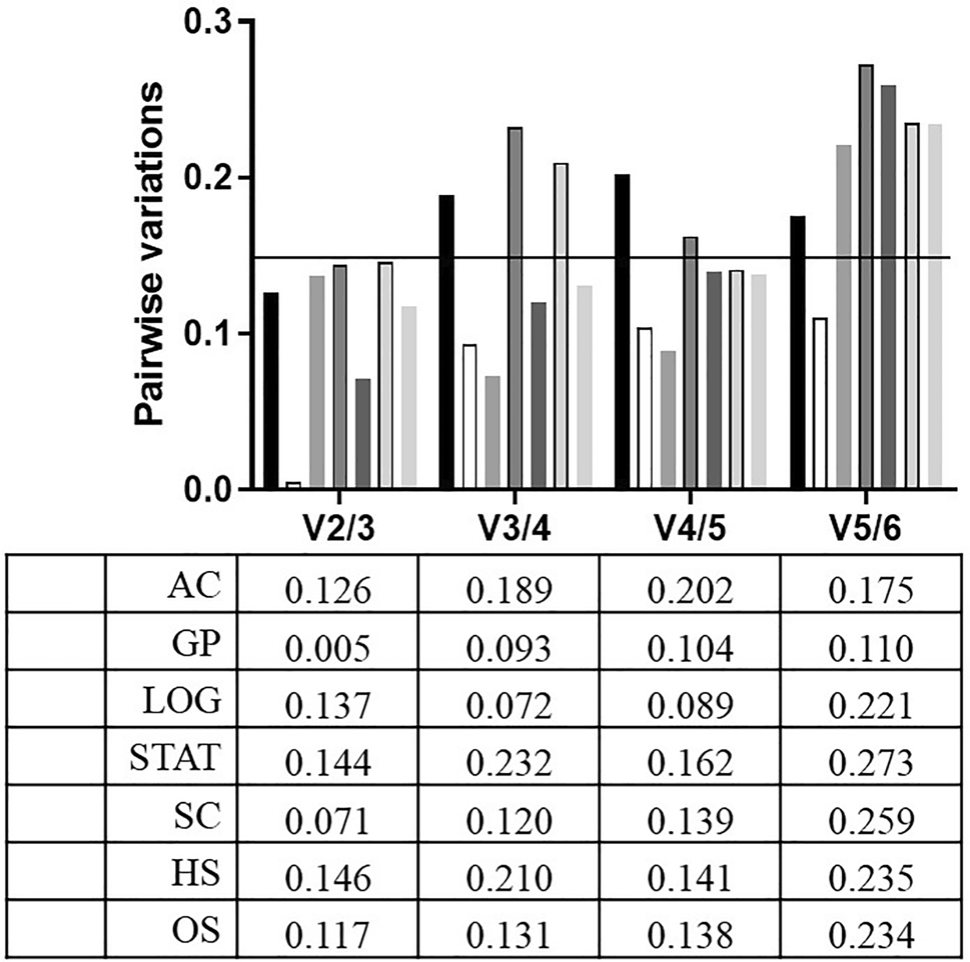
Pairwise variation calculated by geNorm software for *N. gruberi* cultured at different conditions. Vn/Vn + 1 values were used to determine the optimal number of RGs (with threshold value: 0.15). AC: all conditions combined. GP: growth phases: LOG + STAT. SC: stress conditions: HS + OS. LOG: logarithmic phase. STAT: stationary phase. HS: heat shock. OS: oxidative stress.

In addition, geNorm software was used to determine the optimal number of RGs needed for RT-qPCR standardization by calculating the paired variation value Vn/Vn + 1 (n represents the reference gene number). The results reveal the Vn/Vn + 1 values of all the conditions studied were all lower than 0.15 (Fig. 3), suggesting two RGs were sufficient to complete the RT-qPCR normalization in *N. gruberi* under these test conditions.

### Stability of the candidate RGs in different growth stages

The analysis of *N. gruberi* cells at different growth phases (LOG and STAT in combination) revealed the results obtained with the different algorithms were more inconsistent compared with the results obtained when all conditions were analyzed. The genes *G6PD* and *GAPDH*, *HPRT* and *G6PD*, *18S* and *GAPDH,* and *HPRT* and *G6PD* were the two most stable RGs when the analysis was conducted with geNorm, NormFinder, BestKeeper, and RefFinder, respectively (Table 2, Supplementary Table S3).

The analysis of RG stability in trophozoites in LOG phase revealed that NormFinder, BestKeeper, and RefFinder matched for the most stable gene (*TBP*), whereas the most stable genes obtained with geNorm (*18S* and *GAPDH*) did not match any of the previous programs. When the analysis was conducted in STAT trophozoites, geNorm, NormFinder, and RefFinder concurred regarding the most stable genes (*18S*/*GAPDH*), whereas the results of BestKeeper only agreed in one of the genes (*18S*), and the other most stable gene was *G6PD,* which the other algorithms ranked as less stable (Table 2, Supplementary Table S3).

### Stability of the candidate RGs under stress conditions

The stability of the RGs under stress conditions (heat-shock and oxidative stress combined) differed depending on the software employed. When geNorm was used, *18S* and *ACT* genes were the most stable, whereas with NormFinder, *GAPDH* and *G6PD* were the most stable, and the analysis with BestKeeper revealed that *G6PD* and *18S* were the most stable. Finally, the overall ranking according by RefFinder was that *ACT* and, as in the two previous cases, *G6PD* were the most stable RGs (Table 2, Supplementary Table S3).

The most stable genes in heat-shocked trophozoites were common in geNorm, NormFinder, and RefFinder (*TBP*/*G6PD*), but again, BestKeeper agreed only regarding the stability of one of the genes (*TBP*), whereas the most stable gene (*GAPDH*) was considered less stable by the other three programs. When the analysis was conducted with trophozoites under oxidative stress conditions, the RGs were ranked differently in each algorithm: *ACT* and *HPRT*, *G6PD* and *TBP*, *18S* and *G6PD*, *ACT* and *G6PD*, when geNorm, NormFinder, BestKeeper, and RefFinder were respectively used. As in most of the cases studied, the results according to geNorm, NormFinder, and RefFinder were the same for the most and least stable RGs (Table 2, Supplementary Table S3).

The least stable RG results varied for most conditions studied but were identical for stationary cells and heat-shock-treated cells in *HPRT*. Furthermore, when only one condition was analyzed with the four different algorithms, it revealed that geNorm, NormFinder, and RefFinder had the same ranking for the least stable genes, except when all the conditions were combined. Therefore, the results of BestKeeper were not used for validation.

### Validation of the RGs by RT-qPCR

**Figure 4:**
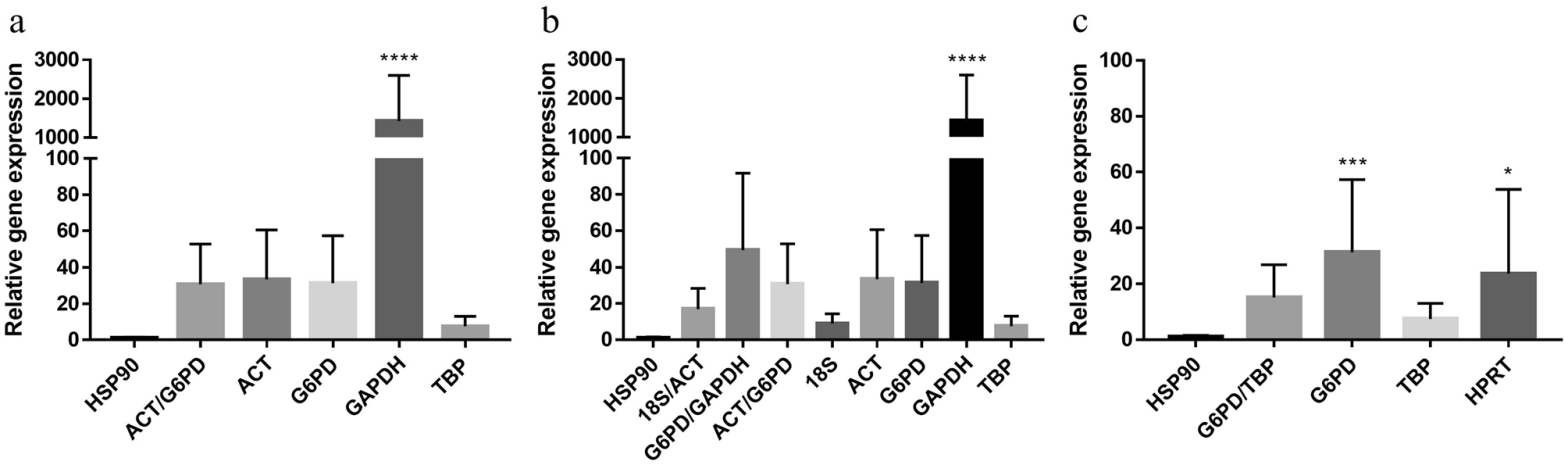
Relative expression of *HSP90* of *N. gruberi* after 1 h heat-shock was compared with the relative expression of *HSP90* of *N. gruberi* control. Normalization with the two most and the least stable RGs. (**a**) Based on results of the analysis of all conditions combined (AC) normalized with the two most stable RGs (*ACT/G6PD*). (**b**) Based on the analysis of stress conditions (SC) normalized with the two most stable RGs (*18S/ACT*, *G6PD/GAPDH* or *ACT/G6PD*). (**c**) Based on the analysis of heat-shock conditions (HS) normalized with the two most stable RGs (*G6PD/TBP*). *p*-values are marked with asterisks (*p < 0.05, **p < 0.01, ***p < 0.001, ****p < 0.0001).

The relative *HSP90* expression of *N. gruberi* was studied after 1 h of heat-shock treatment. Untreated *N. gruberi* cells were used as controls. The relative expression was calculated based on normalization with different RGs for validation. The two most stable RGs, *ACT*/*G6PD* (all conditions in combination) and *G6PD*/*TBP* (after heat-shock), were selected for normalization and were also the most stable RGs under stress conditions. These RGs were different depending on the software used—*18S*/*ACT* (geNorm), *G6PD*/*GAPDH* (NormFinder), and *ACT*/*G6PD* (RefFinder). Moreover, the least stable RGs were employed for normalization, which were *GAPDH* and *TBP* when all conditions were analyzed in combination, *HPRT* under heat-shock, and *TBP* under the combination of stressful conditions. Although *HSP90* relative expression patterns had a similar tendency, which was an increase in expression after the cells were exposed to heat-shock (Fig. 4, Supplementary Tables S5 and S6), normalization with different RGs led to considerable dissimilarities. *HSP90* expression normalized with *ACT*/*G6PD* in combination and alone (Fig. 4a,b) was similar to each other, and no statistical differences were observed. The relative *HSP90* expression was slightly lower if it was normalized with *18S*/*ACT,* the most stable RGs according to geNorm under stressful conditions in combination (Fig. 4b), and *G6PD*/*TBP,* the most stable RGs in agreement with geNorm, NormFinder, and RefFinder following heat-shock treatment (Fig. 4c). *TBP* was considered the least stable RG under all the conditions in combination, displaying low *HSP90* RG expression, so it makes sense the relative expression of *HSP90* expression was also lower when normalized with *G6PD*/*TBP*, leading to an inaccurate *HSP90* relative expression. However, when *GAPDH* also ranked as one of the least stable RGs under all conditions combined, the relative expression of *HSP90* was significantly higher.

Hence, for an appropriate normalization of *HSP90* expression following heat-shock treatment, the use of the combination of *ACT*/*G6PD*, which was suggested by geNorm, NormFinder, and RefFinder as the ideal pair of RGs for *N. gruberi* under all conditions, was the best option.

## Discussion and conclusion

In this study, the stability of different genes was analyzed to determine their appropriateness as RGs for the normalization of gene expression RT-qPCR experiments in *N. gruberi* under different conditions. Choosing the most stable RG to normalize RT-qPCR data is crucial for obtaining the accurate quantification of gene expression and preventing bias by variations that can be introduced from RNA extractions, cDNA synthesis, PCR protocol, and sample loadings^11,13^. In the last decades, genes known as housekeeping genes were broadly used as internal controls for the normalization of RNA levels for Northern blotting, RNAse protection, and RT-qPCR analyses because it was assumed these genes are constitutively expressed and regulated, but no studies have evaluated the stability of expression of these genes under various experimental conditions^11,23^. Moreover, the expression levels of these housekeeping genes vary depending on the conditions and tissues expressed^24–28^. Therefore, it is difficult to select a perfect RG that would be consistently expressed in all tissues and cell types without being influenced by internal or external factors. As a result, recent studies have attempted to determine the stability of RGs under various conditions for several species^29–32^.

However, to date, very few gene expression studies have been conducted for the genus *Naegleria*. In these studies, various genes were used as an internal control, such as β*-actin*^17^, α*-actin*^15^, and *glyceraldehyde 3-phosphate dehydrogenase*^16,18,33^. However, the stability of these genes in the *Naegleria* genus under different conditions is unknown. Interestingly, in this study, the *GAPDH* gene depended on the conditions and algorithm and was not always the most promising choice. Given the lack of a study analyzing the stability of the most commonly used RGs in the genus *Naegleria*, we analyzed these genes in *N. gruberi* as it is a nonpathogenic relative of the brain-eating amoeba. The genes analyzed in this study included those for *18S*, *ACT*, *G6PD*, *GAPDH*, *HPRT,* and *TBP*. This study employed the four widely used programs geNorm^19^, NormFinder^20^, BestKeeper^21^ and RefFinder^22^ on *N. gruberi* trophozites under different conditions. Additionally, the geNorm^19^ algorithm enabled the determination of the number of RGs required for the normalization of gene expression, which in all the conditions investigated for this study was two (Fig. 3). As mentioned in several studies, for accurate and trustworthy results, two or more RGs are needed^19^. Moreover, our findings agree with those found in the genus *Acanthamoeba*, another free-living amoeba, for which geNorm analysis similarly recommended the use of two RGs^34^ and with the results from other protozoan parasites, such as *Trichomonas vaginalis*^35^ and *Trypanosoma brucei*^36^.

The results of this study revealed slight variances among the four programs (Table 2), as reported previously, and these are understandable since each software employs a different algorithm to determine the gene stability^32,37–39^. Moreover, differences in gene stability under the different conditions tested were observed, which were also found in other protozoan parasites^26,34–36^. Therefore, the analysis was also conducted after combining the findings from all the conditions confirming that geNorm, NormFinder, and RefFinder concur regarding the most stable genes (*ACT*/*G6PD*), whereas BestKeeper once more provided a completely different outcome (Table 2). Studies on different organisms have revealed that geNorm, NormFinder, and RefFinder provide similar results, whereas BestKeeper produces a different ranking of gene stability^34,40,41^. This difference can be partially explained since BestKeeper uses raw Ct values as input^21^, whereas relative quantity (RQ) values are used by geNorm^19^ and NormFinder^20^.

To validate that *ACT* and *G6PD* can be used as RGs in all the conditions studied in this work, the expression of *HSP90* in *N. gruberi* trophozoites following 1 h of heat-shock was analyzed, as the expression of this protein is high under heat-shock conditions^34,42–44^ and the relative gene expression obtained when it was normalized with *ACT*/*G6PD* was compared with the one obtained when normalized with *TBP*/*G6PD* (the most stable genes under heat-shock conditions). Our findings demonstrate that fold change values for relative gene expression normalized with *ACT*/*G6PD* are comparable to those normalized with *TBP*/*G6PD* (no statistical difference). *Actin* is the most abundant protein in many eukaryotic cells and is crucial for a wide variety of cellular processes, including cell division, migration, transcriptional regulation, and cell shape regulation, among many others^45,46^. Therefore, *ACT* is widely used as an internal control. This RG revealed greater or lesser expression stability depending on the organism and conditions under study^47^. For instance, the gene is stable in the protozoan human pathogen *T. vaginalis* under nutrient restriction^35^ or in the agricultural pest *Diabrotica undecimpunctata howardi*^29^, but it is not stable in hepatic fibrosis caused by *Schistosoma japonicum*^48^ or in the Siberian giant trout *Hucho taimen* under heat stress^39^. The enzyme *glucose-6-phosphate dehydrogenase* is an important enzyme in all species, from bacteria to mammals, and its metabolic function is to catalyze the first step in the pentose phosphate pathway and to provide the NADPH needed in various biosynthetic and detoxification reactions^49–51^. Similar to *ACT*, *G6PD* is used as an internal control, and its expression varies depending on the organism and condition^34,52,53^. This work, therefore, reaffirms the importance of analyzing the expression stability of RGs on a case-by-case basis.

The use of *N. gruberi* as a safer model of its pathogenic relative *N. fowleri* has been widely established^4,5^. However, since it is not pathogenic, there are biological aspects that cannot be compared between the two species. Nevertheless, the similarity between their genomes is sufficient to make correlations in gene expression studies. Therefore, when conducting gene expression studies in *N. fowleri*, instead of assessing the stability of a wide variety of RGs, the stability of only *ACT* and *G6PD* could be studied and verified regarding stability.

In conclusion, *ACT* and *G6PD* are proposed as reliable RGs for accurate gene expression in *N. gruberi,* as these displayed the best stability when ranked by the different algorithms and under different conditions. Due to the differences in the stability of the RGs in the different conditions studied, it is advisable to review the stability of these genes in other experimental conditions. Moreover, this research revealed that a reliable normalization of gene expression should be conducted with at least two RGs.

## Materials and methods

### Biological samples and experimental conditions

*N. gruberi* NEG-M ATCC 30224 was used as nonpathogenic counterpart for *N. fowleri*^5^. It was cultured at 25 °C in M7 media^54^ in 75 cm^2^ tissue culture flasks with weekly medium changes. Initial experiments were conducted with amoebae in the logarithmic growth phase (LOG cells) and in the stationary growth phase (STAT cells). For logarithmic and stationary cells, 10 ml of amoebae-containing medium from the culture flasks was transferred into fresh flasks and topped up with fresh medium to 20 ml and grown for another 4 days and 14 days, respectively, before harvesting. *N. gruberi* culture in the logarithmic phase was exposed to heat-shock (HS) and oxidative stress (OS). To generate HS cells, growth temperature was shifted for 1 h from 25 to 37 °C, and to generate oxidative stress cells, the cultures were exposed to 250 µM hydrogen peroxide (H_2_O_2_) for 6 h prior to harvesting the cells for RNA isolation. For all the preparations, the flasks were on ice for 10 min to detach the trophozoites, then the amoebae were counted using a Fuchs-Rosenthal counting chamber.

### Selecting the RGs and PCR efficiency study

After referring to general recommendations from other publications^34–36^ and sequences, we chose six candidate RGs for the primer design. The genes selected were the *18S*, *ACT*, *G6PD*, *GAPDH, HPRT, and TBP*. Two pairs of primers non-exon-spanning for each RG were designed using Primer3^55^and were synthesized by Eurofins. The primers were initially tested in conventional PCR using genomic DNA and checked in a 1% agarose gel. Then, using RT-qPCR, standard curves were generated with five points of tenfold serial dilutions of RNA to calculate the primer efficiency (E) and the correlation coefficients (R^2^). Efficiency was calculated according to the formula E = (10^−1/slope^ − 1)*100. The primer pair with the best efficiency in RT-qPCR was selected for further experiments (Table 1).

### RNA extraction and quantitative real-time PCR (RT-qPCR)

Total RNA was isolated from 3 × 10^6^ amoeba using the GeneJET RNA Purification Kit (Thermo Scientific # K0732) following the manufacturer’s protocol. The concentration and purity of RNA were measured using a NanoDrop ND1000 spectrophotometer (NanoDrop Technologies). All the RNA samples were diluted to 10 ng/µl using nuclease-free water and stored at − 80 °C until use.

RT-qPCR was performed in a CFX96 thermocycler (Bio-Rad) using the Luna® Universal One-Step RT-qPCR Kit, which is optimized for the dye-based real-time quantitation of target RNA sequences via the SYBR®/FAM fluorescence channel (New England BioLabs® Inc., E3005L). The reaction mixture (20 µl per reaction) contained 10 µl of Luna Universal One-Step Reaction Mix 2x, 1 µl of Luna WarmStart® RT Enzyme Mix 20x, 400 nM of each primer, and 50 ng of RNA (5 µl of 10 ng/µl). The RT-qPCR profile included a reverse transcription step at 55 °C for 10 min, an initial denaturation step at 95 °C for 1 min, followed by 40 cycles of denaturation at 95 °C for 10 s, and extension at 60 °C for 60 s. A melting curve was performed at the end of the run by stepwise (0.5 °C per 5 s) increasing the temperature from 60 to 95 °C. All the experiments were conducted in three technical and three biological replicates.

### Expression stability analysis of the candidate RGs

The expression stabilities of the RGs in LOG, STAT, HS, and OS cells were individually assessed, and the combination of all these conditions was determined using geNorm^19^, NormFinder^20^ and BestKeeper^21^, which are Microsoft Excel tools, as well as RefFinder^22^, which is a web-based tool. For the geNorm and NormFinder analyses, the raw Cq values were transformed in the RQ using the following formula: RQ = E^(Cq min−Cq sample)^, in which E is the primer efficiency, and Cq min is the lowest Cq value across the sample pool (Supplementary Table S4). Raw Cq values were used directly for BestKeeper and RefFinder. The geNorm program selects the most stable RG by calculating the average expression stability (M-value) of each RG^19^. NormFinder calculates the standard deviation for each gene and compares it with the expression of the other genes. The gene with the lowest variation between intra- and intergroup comparisons is then considered the most stable^20^. BestKeeper reveals stability based on the Pearson coefficient of correlation (r) and standard deviation (SD)^21^. Finally, RefFinder generates a comprehensive ranking by calculating the geometric mean of each RG in the above three methods and delta-Ct method, in which the smaller the ranking, the more stable the RG^22^.

Pair-wise variation (V) was calculated with geNorm to identify the optimal number of RGs required for an accurate normalization for the conditions tested. In this context, the cut-off V value is 0.15, below which the addition of another internal control gene does not result in a significant improvement in normalization.

### Validation of the RG expression

The relative gene expression of the target gene *HSP90* was analyzed using the Pfaffl method^56^, employing all the RGs separately for normalization. Additionally, based on pair-wise variation, expression patterns of the target gene were normalized with the two most stable RGs using the Vandesompele method^19^. The relative change of expression upon normalization was compared with the calculated RQ of the target gene based on the formula RQ = E^−ΔCq^ by comparing the Cq values of LOG cells with the HS cells. This calculation was based on each RT-qPCR having the same amount of RNA.

### Statistical analysis

Statistical analysis was performed with GraphPad Prism 8 (GraphPad Software Inc., USA). To determine statistical significance among investigated groups, one-way analysis of variance (ANOVA) was performed. Statistical difference was considered when *p* < 0.05.

### Supplementary Information

Supplementary Information.

## Data Availability

All data generated or analyzed during this study are included in this published article [and its supplementary information files].
