# Supplementary material for: Evaluation and validation of reference genes for RT-qPCR gene expression in Naegleria gruberi
Source: Sci Rep. 2023 Oct 5;13:16748. doi: 10.1038/s41598-023-43892-3 (PMC10555999; doi:10.1038/s41598-023-43892-3)
Supplement: Supplementary file 1 — Supplementary Information. [file 41598_2023_43892_MOESM1_ESM.docx]

**Evaluation and validation of reference genes for RT-qPCR gene expression in *Naegleria gruberi***

Tania Martín-Pérez*, Martina Köhsler and Julia Walochnik

Institute of Specific Prophylaxis and Tropical Medicine, Center for Pathophysiology, Infectiology and Immunology, Medical University of Vienna, 1090 Vienna, Austria

*corresponding author

**Supplementary Figure S1.** The specificities of the primers pairs for qRT-PCR amplification. The PCR product of each gene was confirmed using 1% agarose gel. Lane 1: *18S* (1); Lane 2: *18S* (2); Lane 3: *HPRT* (2); Lane 4: *HPRT* (1); Lane 5: *GAPDH* (1); Lane 6: *GAPDH* (2); Lane 7: *ACT* (2); Lane 8: *ACT* (1); Lane 9: *TBP* (2); Lane 10: *TBP* (1); Lane 11: *G6PD* (2); Lane 12: *G6PD* (1).


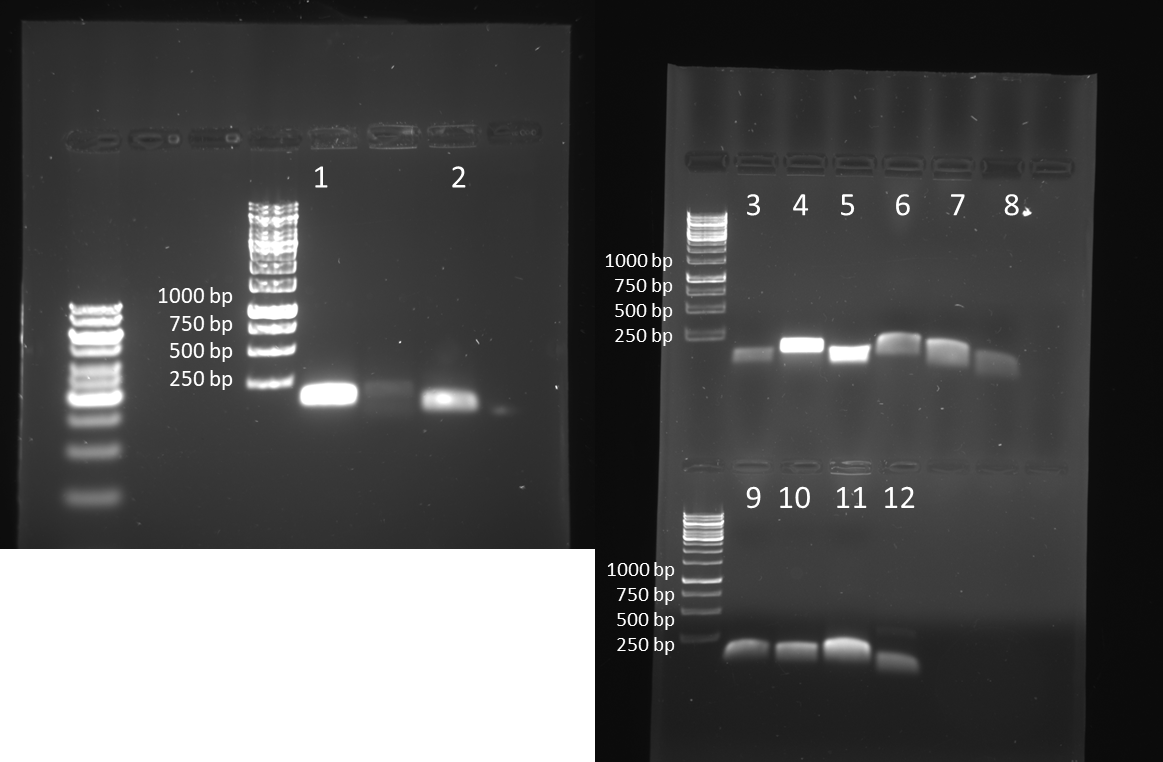


**Supplementary Table S1**. Mean Cq values of all RGs in all conditions tested: LOG: logarithmic phase cultures. STAT: stationary phase cultures. HS: heat shock cultures. OS: oxidative stress with H_2_O_2_. Mean control and tested Ct values of gen of interest (HSP90).

| Gene | LOG | STAT | HS | OS |
| --- | --- | --- | --- | --- |
| *18S* | 6,578 | 5,712 | 4,439 | 4,034 |
| *ACT* | 24,441 | 24,813 | 24,078 | 23,773 |
| *G6PD* | 20,217 | 20,562 | 19,585 | 19,533 |
| *GAPDH* | 14,767 | 15,136 | 15,316 | 15,871 |
| *HPRT* | 23,155 | 23,006 | 22,854 | 21,949 |
| *TBP* | 20,220 | 19,728 | 17,637 | 19,619 |
| *HSP90* | 21,377 |  | 21,164 |  |

**Supplementary Table S2**. Mean Cq values after efficiency correction (CqE) of all RGs in all conditions tested: LOG: logarithmic phase cultures. STAT: stationary phase cultures. HS: heat shock cultures. OS: oxidative stress with H_2_O_2_. Mean control and tested Ct values of gen of interest (HSP90).

| Gene | LOG | STAT | HS | OS |
| --- | --- | --- | --- | --- |
| *18S* | 6,384 | 5,576 | 4,309 | 3,928 |
| *ACT* | 25,474 | 25,862 | 25,097 | 24,778 |
| *G6PD* | 21,251 | 21,614 | 20,587 | 20,532 |
| *GAPDH* | 14,909 | 15,282 | 15,464 | 16,024 |
| *HPRT* | 23,889 | 23,735 | 23,579 | 22,645 |
| *TBP* | 20,138 | 19,648 | 17,566 | 19,539 |
| *HSP90* | 20,748 |  | 21,192 |  |

**Supplementary Table S3**. Expression stability values for all RGs calculated with all algorithms and all conditions tested. (AC: all conditions. GP: LOG/STAT cultures. LOG: logarithmic phase cultures. STAT: stationary phase cultures. SC: stressful conditions. HS: heat shock cultures. OS: oxidative stress with H_2_O_2_).

| geNorm | | | | | | | | | | | | | | |
| --- | --- | --- | --- | --- | --- | --- | --- | --- | --- | --- | --- | --- | --- | --- |
| Gene | Expression stability M | | | | | | | Ranking | | | | | | |
|  | AC | GP | LOG | STAT | SC | HS | OS | AC | GP | SC | LOG | STAT | HS | OS |
| *18S* | 0.539 | 0.436 | 0.003 | 0.129 | 0.053 | 0.621 | 0.424 | 4 | 6 | 1 | 1 | 1 | 4 | 4 |
| *ACT* | 0.136 | 0.012 | 0.688 | 0.639 | 0.053 | 0.689 | 0.223 | 1 | 3 | 1 | 6 | 4 | 5 | 1 |
| *G6PD* | 0.136 | 0.007 | 0.297 | 0.743 | 0.160 | 0.312 | 0.310 | 1 | 1 | 3 | 4 | 5 | 1 | 3 |
| *GAPDH* | 0.881 | 0.007 | 0.003 | 0.129 | 0.470 | 0.400 | 0.839 | 6 | 1 | 5 | 1 | 1 | 3 | 6 |
| *HPRT* | 0.299 | 0.193 | 0.366 | 1.045 | 0.320 | 0.951 | 0.223 | 3 | 4 | 4 | 5 | 6 | 6 | 1 |
| *TBP* | 0.750 | 0.323 | 0.276 | 0.331 | 0.832 | 0.312 | 0.549 | 5 | 5 | 6 | 3 | 3 | 1 | 5 |
| NormFinder | | | | | | | | | | | | | | |
| Gene | Stability value | | | | | | | Ranking | | | | | | |
|  | AC | GP | LOG | STAT | SC | HS | OS | AC | GP | SC | LOG | STAT | HS | OS |
| *18S* | 0.518 | 0.450 | 0.365 | 0.045 | 0.199 | 0.558 | 0.421 | 4 | 6 | 4 | 5 | 1 | 5 | 4 |
| *ACT* | 0.109 | 0.237 | 0.914 | 0.649 | 0.112 | 0.379 | 0.200 | 1 | 5 | 3 | 6 | 4 | 3 | 3 |
| *G6PD* | 0.109 | 0.219 | 0.039 | 0.711 | 0.065 | 0.259 | 0.088 | 1 | 2 | 2 | 1 | 5 | 2 | 1 |
| *GAPDH* | 0.569 | 0.226 | 0.363 | 0.045 | 0.035 | 0.379 | 0.954 | 5 | 4 | 1 | 4 | 1 | 3 | 6 |
| *HPRT* | 0.307 | 0.083 | 0.042 | 1.120 | 0.593 | 0.964 | 0.435 | 3 | 1 | 5 | 3 | 6 | 6 | 5 |
| *TBP* | 0.592 | 0.224 | 0.039 | 0.129 | 1.072 | 0.108 | 0.132 | 6 | 3 | 6 | 1 | 3 | 1 | 2 |
| BestKeeper | | | | | | | | | | | | | | |
| Gene | Coefficient correlation | | | | | | | Ranking | | | | | | |
|  | AC | GP | LOG | STAT | SC | HS | OS | AC | GP | SC | LOG | STAT | HS | OS |
| *18S* | 0.934 | 0.842 | 0.939 | 0.986 | 0.503 | 0.712 | 0.895 | 1 | 1 | 2 | 3 | 2 | 4 | 1 |
| *ACT* | 0.425 | 0.074 | 0.985 | 0.630 | 0.053 | 0.004 | 0.053 | 5 | 6 | 6 | 2 | 6 | 6 | 6 |
| *G6PD* | 0.663 | 0.556 | 0.919 | 0.993 | 0.782 | 0.942 | 0.881 | 3 | 4 | 1 | 5 | 1 | 3 | 2 |
| *GAPDH* | 0.318 | 0.685 | 0.935 | 0.907 | 0.363 | 0.994 | 0.067 | 6 | 2 | 3 | 4 | 5 | 1 | 5 |
| *HPRT* | 0.446 | 0.454 | 0.646 | 0.944 | 0.329 | 0.685 | 0.291 | 4 | 5 | 4 | 6 | 4 | 5 | 4 |
| *TBP* | 0.668 | 0.678 | 1.001 | 0.967 | 0.135 | 0.953 | 0.348 | 2 | 3 | 5 | 1 | 3 | 2 | 3 |
| RefFinder | | | | | | | | | | | | | | |
| Gene | Geomean of ranking values | | | | | | | Ranking | | | | | | |
|  | AC | GP | LOG | STAT | SC | HS | OS | AC | GP | SC | LOG | STAT | HS | OS |
| *18S* | 4.43 | 6 | 3.16 | 1.73 | 2.45 | 3.16 | 4.73 | 5 | 6 | 3 | 4 | 2 | 3 | 5 |
| *ACT* | 1.41 | 2.99 | 6 | 4 | 1.41 | 3.31 | 1.57 | 1 | 4 | 1 | 6 | 4 | 4 | 1 |
| *G6PD* | 1.68 | 2.21 | 1.86 | 5 | 1.57 | 2 | 1.57 | 2 | 2 | 2 | 2 | 5 | 2 | 1 |
| *GAPDH* | 3.83 | 2.45 | 2.63 | 1.19 | 3.94 | 3.66 | 6 | 4 | 3 | 4 | 3 | 1 | 5 | 6 |
| *HPRT* | 3 | 1.41 | 3.87 | 6 | 4.73 | 6 | 2.63 | 3 | 1 | 5 | 5 | 6 | 6 | 3 |
| *TBP* | 5 | 4.40 | 1.68 | 2.45 | 6 | 1.32 | 3.31 | 6 | 5 | 6 | 1 | 3 | 1 | 4 |

**Supplementary Table S4**. Relative quantities (RQ) used for geNorm and NormFinder analysis. (AC: all conditions. GP: LOG/STAT cultures. LOG: logarithmic phase cultures. STAT: stationary phase cultures. SC: stressful conditions. HS: heat shock cultures. OS: oxidative stress with H_2_O_2_).

| **AC** | | | | | | |
| --- | --- | --- | --- | --- | --- | --- |
|  | *18S* | *HPRT* | *ACT* | *G6PD* | *GAPDH* | *TBP* |
| LOG | 0.180576 | 0.422301 | 0.617294 | 0.607403 | 1 | 0.168099 |
| STAT | 0.323364 | 0.469786 | 0.471794 | 0.472551 | 0.772421 | 0.236166 |
| HS | 0.761176 | 0.523505 | 0.801907 | 0.962896 | 0.680896 | 1 |
| OS | 1 | 1 | 1 | 1 | 0.461845 | 0.254688 |
| **GP** | | | | | | |
|  | 18S | HPRT | ACT | G6PD | GAPDH | TBP |
| LOG | 0.55843 | 0.898921 | 1 | 1 | 1 | 0.711783 |
| STAT | 1 | 1 | 0.764293 | 0.777985 | 0.772421 | 1 |
| **LOG** | | | | | | |
|  | *18S* | *HPRT* | *ACT* | *G6PD* | *GAPDH* | *TBP* |
| Biological replicate 1 | 1 | 0.622339 | 0.158389 | 0.74705 | 1 | 0.788177 |
| Biological replicate 2 | 0.844191 | 0.633989 | 0.56828 | 1 | 0.84408 | 0.936856 |
| Biological replicate 3 | 0.71837 | 1 | 1 | 0.921784 | 0.721009 | 1 |
| **STAT** | | | | | | |
|  | *18S* | *HPRT* | *ACT* | *G6PD* | *GAPDH* | *TBP* |
| Biological replicate 1 | 1 | 1 | 0.335801 | 0.320377 | 1 | 0.735964 |
| Biological replicate 2 | 0.711476 | 0.15518 | 1 | 1 | 0.796399 | 0.92989 |
| Biological replicate 3 | 0.740613 | 0.298788 | 0.392856 | 0.87823 | 0.88396 | 1 |
| **SC** | | | | | | |
|  | *18S* | *HPRT* | *ACT* | *G6PD* | *GAPDH* | *TBP* |
| HS | 0.761176 | 0.523505 | 0.801907 | 0.962896 | 1 | 1 |
| OS | 1 | 1 | 1 | 1 | 0.678289 | 0.254688 |
| **HS** | | | | | | |
|  | *18S* | *HPRT* | *ACT* | *G6PD* | *GAPDH* | *TBP* |
| Biological replicate 1 | 0.728393 | 1 | 0.916161 | 1 | 1 | 1 |
| Biological replicate 2 | 1 | 0.11072 | 0.620192 | 0.712458 | 0.483177 | 0.810181 |
| Biological replicate 3 | 0.878215 | 0.295711 | 1 | 0.376033 | 0.261278 | 0.573152 |
| **OS** | | | | | | |
|  | *18S* | *HPRT* | *ACT* | *G6PD* | *GAPDH* | *TBP* |
| Biological replicate 1 | 0.424791 | 0.561947 | 0.740116 | 0.681859 | 1 | 1 |
| Biological replicate 2 | 1 | 0.70734 | 0.915852 | 1 | 0.348115 | 0.804679 |
| Biological replicate 3 | 0.543219 | 1 | 1 | 0.769294 | 0.171357 | 0.534305 |

**Supplementary Table S5**. Calculated gene expression ratio target gene (*HSP90*) based on Vandesompele normalization method with a combination of the two best ranked RGs under all the conditions combined (AC), under stress conditions (SC) and under heat-shock conditions (HS). (GN: geNorm, NF: NormFinder, RF: RefFinder).

| AC | | | SC | | | HS | | |
| --- | --- | --- | --- | --- | --- | --- | --- | --- |
| Algorithm | best two RGs | Relative gene expression | Algorithm | best two RGs | fold change | Algorithm | best two RGs | Relative gene expression |
| GN/NF/RF | *ACT/G6PD* | 30.71 | GN | *18S/ACT* | 16.86 | GN/NF/RF | *G6PD/TBP* | 15.09 |
|  |  |  | NF | *G6PD/GAPDH* | 49.54 |  |  |  |
|  |  |  | RF | *ACT/G6PD* | 30.71 |  |  |  |
|  |  |  |  |  |  |  |  |  |
|  |  |  |  |  |  |  |  |  |

**Supplementary Table S6**. Calculated gene expression ratio target gene (*HSP90*) based on Pfaffl normalization method for all RGs independently.

| RG | Relative gene expression |
| --- | --- |
| *HSP90* | **1.01** |
| *18S* | 8.91 |
| *ACT* | 33.41 |
| *G6PD* | 31.35 |
| *GAPDH* | 1,421.94 |
| *HPRT* | 61.73 |
| *TBP* | 7.48 |
